# Artificial intelligence for early diagnosis of mild cognitive impairment: A scoping review comparing diagnostic accuracy with physician performance

**DOI:** 10.1097/MD.0000000000050006

**Published:** 2026-07-31

**Authors:** Haewon Byeon

**Affiliations:** a Department of Future Technology, Korea University of Technology and Education, Cheonan, South Korea.

**Keywords:** artificial intelligence, deep learning, diagnosis, machine learning, mild cognitive impairment, physician performance, scoping review

## Abstract

**Background:**

Mild cognitive impairment (MCI) represents a clinically critical and heterogeneous syndrome characterized by cognitive decline exceeding normal aging expectations, yet insufficient to impair daily functioning. As the prodromal stage of dementia, MCI offers a crucial intervention window during which therapeutic strategies can modify disease trajectory. Therefore, accurate and early diagnosis is of paramount importance. Artificial intelligence (AI) systems have demonstrated promising performance across multiple diagnostic modalities, yet their performance relative to that of physicians remains incompletely characterized in the literature.

**Methods:**

A systematic search of 6 electronic databases (PubMed/MEDLINE, EMBASE, Web of Science, Scopus, PsycINFO, and CINAHL) was conducted, resulting in 1358 records. Following de-duplication, systematic evaluation of abstracts and full text applying prespecified eligibility criteria, and exclusion of systematic reviews and meta-analyses, 71 primary research studies were included for analysis.

**Results:**

The included studies comprised 71 primary research studies published between 2008 and 2026. AI systems achieved diagnostic accuracy ranging from 62% to 100%, with neuroimaging-based and multimodal approaches often reporting the strongest performance (85–99%). Only 8 studies directly compared AI with physician diagnostic performance. In these limited and methodologically heterogeneous comparisons, AI systems generally matched or exceeded physician performance, although the available evidence base remains small. An artificial neural network achieved 90.0% sensitivity and 84.78% specificity compared to a panel of physicians collectively 46.66% sensitivity, while GPT-4 outperformed junior neurologists in 1 language-based study (81% vs 41–49%; *P* < .001). AI assistance improved neurologist diagnostic performance in 1 large study by approximately 26% in area under the receiver operating characteristic curve (AUROC) (*P* < .05). Most studies used internal validation without external datasets, limiting conclusions about real-world generalisability.

**Conclusion:**

AI approaches for early diagnosis of MCI appear promising and may support or enhance physician performance in selected settings, but widespread clinical adoption requires prospective validation, standardized physician benchmarking, and rigorous evaluation in real-world clinical environments.

## 1. Introduction

Dementia currently affects more than 55 million people worldwide, with a prevalence projected to increase to approximately 153 million by 2050 as populations age globally.^[1]^ Mild cognitive impairment (MCI) occupies the transition zone between normal cognitive aging and frank dementia, characterized by subjective and objectively measurable cognitive decline in 1 or more domains, most commonly memory – that does not substantially interfere with daily functioning.^[2]^

The clinical importance of MCI lies primarily in its role as an intervention window: approximately 10% to 15% of people with MCI progress to Alzheimer disease annually, compared to 1% to 2% of the general population.^[3]^ Therefore, an accurate diagnosis of MCI has profound implications for treatment allocation, clinical trial enrollment, and patient counseling. However, the diagnosis of MCI remains clinically challenging, mainly due to its heterogeneity in etiology, presentation, and trajectory. Standard evaluation combines validated cognitive screening tools – most prominently the Mini-Mental State Examination (MMSE) and the Montreal Cognitive Assessment (MoCA) – with neuropsychological testing, neuroimaging, and biomarker analysis. Despite this multifaceted approach, diagnostic accuracy for MCI remains substantially lower than that for established dementia. In one head-to-head study, a panel of 4 physicians achieved a collective sensitivity of 46.66% for identifying MCI, whereas an artificial neural network (ANN) achieved 90.0% sensitivity in the same sample.^[4]^ Similarly, neuroradiologists reviewing structural magnetic resonance imaging (MRI) achieved diagnostic accuracies of 57.5% to 70.0%, below the 90.5% accuracy reported for an AI classifier in the same study.^[5]^

AI systems, which encompass machine learning, deep learning, and natural language processing approaches, have emerged as promising tools for early diagnosis of MCI across multiple data modalities. Some recent studies have reported favorable AI performance compared with specific physician groups in selected tasks. In one individual language-based classification study, GPT-4 showed higher diagnostic accuracy than junior neurologists (*P* < .001),^[6]^ while transformer-based architectures that integrate multimodal clinical data in 51,269 participants achieved a 26% improvement in diagnostic AUROC when used to support clinical assessment compared with unassisted evaluation.^[7]^ Additional high-performance primary studies have reported excellent diagnostic accuracy across modalities, including an ensemble model using demographic and biomarker data that achieved an accuracy of 83.2%,^[8]^ a virtual kiosk approach using hand and eye movement analysis that achieved a sensitivity of 93.3%,^[9]^ and a DenseNet169 transfer learning model applied to structural MRI that achieved a precision of 99.7%.^[10]^

Despite this growing evidence base, the comparative literature between AI systems and physicians remains sparse and methodologically heterogeneous. The purpose of this scoping review is to systematically map and synthesize evidence on AI-based diagnostic accuracy for MCI, with particular emphasis on direct comparisons with physician performance. Specific objectives are: to characterize the AI methodologies, input data modalities, and clinical contexts used in MCI diagnosis studies; to summarize AI diagnostic performance in studies and modalities; to describe direct comparative evidence between AI and physician diagnostic performance; examine clinical implementation considerations; and identify priorities for future research and clinical translation.

## 2. Materials and methods

This scoping review was conducted and reported in accordance with the PRISMA-ScR framework.

### 2.1. Search strategy

A systematic search of the literature was conducted in 6 electronic databases: PubMed/MEDLINE, EMBASE, Web of Science, Scopus, PsycINFO, and CINAHL. The search covered publications from January 2000 to March 2026 and was restricted to English-language articles. The search strategy combined medical subject headings (MeSH terms) and free-text keywords organized around 3 core concept domains: mild cognitive impairment; artificial intelligence and machine learning; and diagnostic precision. Boolean operators (AND, OR) were used to combine terms across and within domains. The reference lists of the included articles were hand searched for additional eligible studies. A total of 1358 records were identified. The full database-specific search strings, including Boolean operators and search fields, are provided in Supplementary Appendix 1, Supplemental Digital Content 1.

### 2.2. Eligibility criteria

Studies were eligible for inclusion if they: involved patients with suspected MCI or individuals at risk of cognitive decline, using validated diagnostic criteria such as the Petersen criteria or NIA-AA guidelines; evaluated AI-based diagnostic tools, algorithms, or machine learning methods specifically for MCI diagnosis; reported quantitative diagnostic performance measures, including sensitivity, specificity, general accuracy, area under the curve (AUC), F1-score, positive predictive value (PPV), or negative predictive value (NPV); and were original primary research articles. Studies were excluded if they were systematic reviews, meta-analyses, case reports, case series, editorials, or conference abstracts lacking complete methodology; focused exclusively on dementia without a specific MCI diagnostic component; or provided insufficient data to extract meaningful diagnostic performance measures.

### 2.3. Screening process

**Figure 1.**
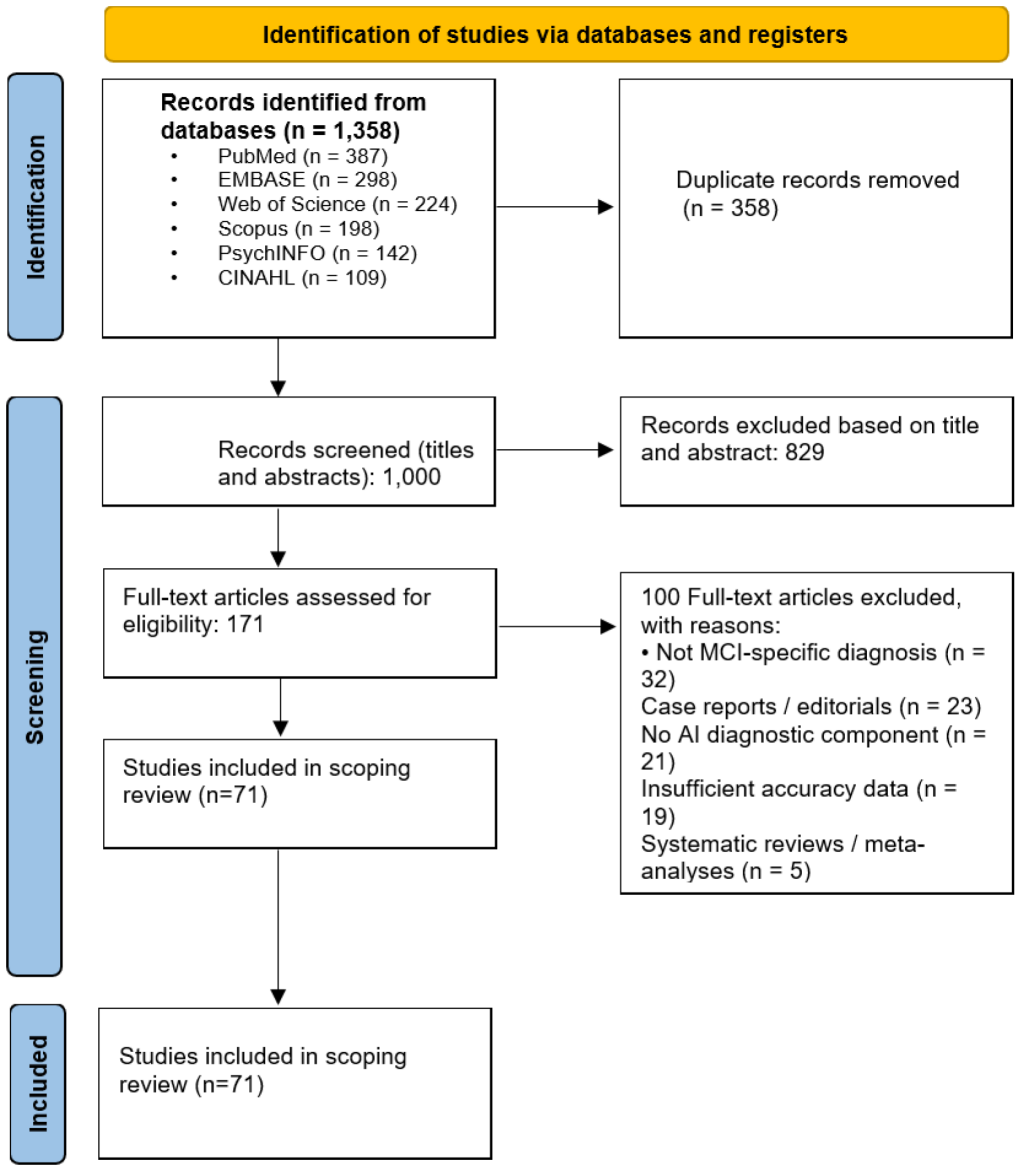
PRISMA-ScR flow diagram of study selection. MCI = mild cognitive impairment.

After removing 358 duplicate records, a total of 1000 records underwent title and abstract screening. At this stage, 829 records were excluded for failure to meet 1 or more inclusion criteria. The remaining 171 records were evaluated in full text for eligibility. Of the 171 full texts reviewed, 100 were excluded: no MCI-specific diagnosis (n = 32), no AI diagnostic component (n = 21), insufficient diagnostic performance data for extraction (n = 19), case reports, editorials, or conference abstracts without full methodology (n = 23), and systematic reviews or meta-analyses (n = 5). A total of 71 primary research studies were included in the final analysis. Figure 1 shows the PRISMA-ScR study selection flow.

### 2.4. Data extraction

Data extraction was performed using a structured template that captured: details of the AI method, including algorithm type, architecture, and input data types; diagnostic performance, including sensitivity, specificity, AUC, general accuracy, F1-score, PPV, and NPV when reported; physician performance data where available; characteristics of the study population; validation approach; clinical context; and comparative analysis between AI and physician performance when reported.

### 2.5. Analysis

Data were collated and synthesized using a descriptive approach consistent with scoping review methodology. Quantitative performance metrics were organized by AI methodology, input data modality, clinical context, validation strategy, and study scale to identify consistent patterns and sources of heterogeneity. Narrative synthesis was used to integrate findings. No formal meta-analysis was performed due to substantial heterogeneity in study designs, populations, AI methodologies, and outcomes. No formal risk-of-bias assessment was undertaken because the aim of this scoping review was evidence mapping rather than pooled effect estimation; however, methodological limitations relevant to interpretation – such as sample size, retrospective design, internal-only validation, and restricted generalisability – were examined narratively. No prior protocol registration in PROSPERO or OSF was undertaken for this scoping review.

## 3. Results

### 3.1. Study selection

The initial search in 6 electronic databases (PubMed/MEDLINE, EMBASE, Web of Science, Scopus, PsychINFO, and CINAHL) identified a total of 1358 records. After removing 358 duplicate entries, 1000 unique records remained for selection. During the title and abstract screening phase, 829 records were excluded as they did not meet the preliminary inclusion criteria. Consequently, 171 full-text articles were recovered and assessed for eligibility. Of these, 100 articles were excluded for the following reasons: lack of a MCI-specific diagnosis (n = 32), inclusion of case reports or editorials (n = 23), absence of an AI diagnostic component (n = 21), insufficient precision data (n = 19), or being systematic reviews/meta-analyses (n = 5). Ultimately, 71 primary research studies met all inclusion criteria and were included in the final scoping review. Studies were published between 2008 and 2026, with most (87%) published after 2020. Figure 1 presents the final PRISMA-ScR flow diagram.

### 3.2. Characteristics of included studies

The 71 included studies covered a wide array of AI methodologies and input data types. Study populations ranged from as few as 18 participants in small feasibility studies^[11,12]^ to 51,269 participants in large-scale retrospective analyses.^[7]^ Most primary studies enrolled between 50 and 500 participants. Large-scale studies were represented by a multimodal deep learning approach in 8916 participants,^[13]^ a multi-algorithm clinical questionnaire-based study in 5272 hospitalized individuals,^[14]^ and a large dementia center screening study involving 14,000 patients.^[15]^ Table 1 summarizes the characteristics of all 71 included studies.

**Table 1:** Characteristics of all included studies.

Study	AI method	Input data	Setting	Study population
[4]	Counterpropagation ANN	MMSE, FAQ, GDS, demographics	Primary care	128 MCI, 203 controls
[5]	SVM with VBM (BAAD/VSRAD)	Structural MRI, MMSE	Primary/specialty care	1446 subjects
[6]	GPT-4	Language samples	Research setting	174 participants
[7]	Transformer (multimodal)	Imaging + clinical data	Primary/specialty care	51,269 participants
[8]	Ensemble (Extra Trees, RF, LightGBM)	Age, gender, biomarkers	Primary care/screening	1972 subjects
[9]	SVM	Virtual kiosk test (hand/eye movement)	Primary care/screening	51 participants
[10]	DenseNet169 transfer learning	Structural MRI	Research center	398 participants
[11]	Multiple ML algorithms	Game-based cognitive tasks	Community/primary care	30 subjects
[12]	SVM, Random Forests	Mini-SEA, MMSE, EEG	Research/specialty	18 participants
[13]	CNN + CatBoost (multimodal)	Magnetic resonance imaging, demographics, neuropsych tests	Multiple settings	8916 participants
[14]	Multiple ML algorithms	Clinical questionnaire	Hospital setting	5272 individuals
[15]	SVM, Naive Bayes, MLP	MMSE-KC, CERAD-K	Dementia center	14,000 patients
[16]	Ensemble classifier	Structural MRI	Research context	1101 subjects
[17]	KNN, SVM, RF, DT, MLP	2D MRI, clinical features	Specialty/research	400 subjects
[18]	SVM	Linguistic variables (CERAD, WMS)	Specialty clinics	68 MCI, 304 controls
[19]	Binary logistic regression	ICA test responses	Primary care/screening	230 participants
[20]	SVM	Structural MRI	Research center	30 subjects
[21]	SVM, Random Forest	Resting-state fMRI	Specialty/research	78 participants
[22]	SVM	EEG, eye tracking, neuropsych	Community screening	428 participants
[23]	XGBoost	MRI, MMSE, CDR	Specialty/research	663 subjects
[24]	SVM, Random Forest	Electronic medical records	Primary care	283 patients
[25]	Weighted soft voting (ensemble)	Digital cognitive tasks, physiological	Community/home use	120 participants
[26]	SVM, Extra Trees, RF, LR, DT	Speech-based digital assessment	Memory clinics/screening	190 participants
[27]	CNN with MKSCDDL kernel	MRI, PET	Specialty/research	12,000 MRI slices
[28]	SVM, pSVM	PiB-PET scans	Specialty/screening	84 scans
[29]	Multiple ML algorithms	MMSE, MoCA, KDSQ	Specialty clinic	955 participants
[30]	XGBoost, SVM, KNN	MRI	Specialty/hospital	158 participants
[31]	2D CNN	Structural MRI	Specialty/screening	3312 images
[32]	CNN with self-attention	CDT, cube-copying, trail-making	Specialty clinic	918 subjects
[33]	3D CNN, fuzzy clustering	MRI, CSF biomarkers	Primary care/screening	630 MRI images
[34]	DAG 3D-CNN with SVM	Structural MRI	Specialty/research	455 participants
[35]	Ensemble CNNs	EEG connectivity patterns	Primary care/screening	187 participants
[36]	CNN	fNIRS spatial features	Specialty/research	230 subjects
[37]	Dual-branch CNN	EEG spectrograms, cube-drawing	Specialty clinic	114 participants
[38]	Gradient Boosting	Structured EHR data	Primary care/community	~640 patients (test)
[39]	CNN, Deep Metric Learning	MRI	Specialty/hospital	578 participants
[40]	SVM, RF, MLP, CNN	Gait, body composition, sleep, MRI	Community/screening	80 patients
[41]	CNN	3D T1-weighted MRI	Specialty/research	1638 subjects
[42]	Ensemble of SVMs	Magnetic resonance imaging, neuropsychological tests	Memory clinics	426 patients
[43]	RF, XGBoost, LightGBM, CatBoost	ReCOGnAIze app, MRI	Primary care/community	235 participants
[44]	Deep Neural Networks	Speech acoustic features	Primary care/screening	55 participants
[45]	NetraAI platform (multimodal)	Digital tasks, multimodal	Research center	98 participants
[46]	Random Forest	TMS measures	Specialty setting	160 participants
[47]	BERT, ensemble learning	Written picture descriptions	Specialty clinic	169 individuals
[48]	BERT, ensemble learning	Written picture descriptions	Specialty clinic	169 individuals
[49]	Random Forest	Demographics, clinical data, MMSE	Primary healthcare	763 participants
[50]	SVM (multimodal)	VR biomarkers, MRI	Primary care/screening	54 participants
[51]	Logistic regression, RF	MRI radiomics, neuropsych, plasma	Specialty/screening	268 participants
[52]	LASSO logistic regression	Clinical notes (NLP)	Primary care	4185 patients
[53]	XGBoost	Advanced dMRI, CSF biomarkers	Specialty/research	189 subjects
[54]	Spectral Clustering, K-Means, GMM	Neuropsych, clinical data	Research center	115 PwPD, 226 controls
[55]	Binary logistic regression	ICA test	Primary care	99 participants
[56]	MKSCDDL	sMRI, FDG-PET, florbetapir-PET	Research center	340 subjects
[57]	Deep learning (fully connected NN)	MMSE item scores	Clinical setting	164 participants
[58]	PredictAD tool	MRI, CSF, cognitive tests	Specialty clinic	391 MCI cases
[59]	Logistic Regression, KNN	OCAT eye movement features	Clinic/bedside/remote	206 participants
[60]	Logistic regression, SVM	Speech analysis, Cookie Theft task	Specialty clinic	185 participants
[61]	LASSO regression	CBF predictions, neuropsych tests	Specialty clinic	103 participants
[62]	Multilayer perceptron ANN	T1-weighted MRI	Research center	110 participants
[63]	LDA, QDA	Cognitive tests, sMRI, RS-fMRI	Specialty clinic	100 subjects
[64]	Multi-layer Perceptron	Neuropsych tests, demographics	Specialty/primary care	1937 patients
[65]	Multilayer perceptron	5-min cognitive task	Primary care	57 participants
[66]	Random Forest	MRI, neuropsych, lab tests	Specialty/hospital	90 participants
[67]	Multi Classifier System	MRI-based features, MMSE	Research center	Not detailed
[68]	LightGBM, ensemble methods	fNIRS, neuropsych tests	Primary care/screening	133 participants
[69]	Machine learning classifiers	Speech recordings	Primary/specialty care	165 participants
[70]	Deep learning (hierarchical attention)	Clinical notes from EHR	Primary care/health system	2166 patients
[71]	Deep Neural Network	Plasma proteomic biomarkers	Research center	239 adults
[72]	KNN, LR, SVM, CNN	Facial emotion analysis	Specialty clinic	64 participants
[73]	CNN, k-NN, LR, RF	Keystroke dynamics, linguistic	Nonclinical/community	23 participants
[74]	LASSO, SVM	Urine proteomics	Specialty clinic	162 participants

AI = artificial intelligence, ANN *= *artificial neural network, CBF *= *cerebral blood flow, CDR *= *Clinical Dementia Rating, CDT *= *clock drawing test, CNN *= *convolutional neural network, CSF *= *cerebrospinal fluid, DT *= *decision tree, EEG *= *electroencephalography, EHR *= *electronic health record, FAQ *= *Functional Activities Questionnaire, fMRI *= *functional magnetic resonance imaging, fNIRS *= *functional near-infrared spectroscopy, GDS *= *Geriatric Depression Scale, GMM *= *Gaussian mixture model, KNN *= *k-nearest neighbor, LASSO *=* least absolute shrinkage and selection operator, LDA *=* linear discriminant analysis, LR *= *logistic regression, MCI *= *mild cognitive impairment, MKSCDDL *= *multi-kernel sparse coding discriminant dictionary learning, MLP *=* multilayer perceptron, MMSE *=* Mini-Mental State Examination, MoCA *= *Montreal Cognitive Assessment, MRI *= *magnetic resonance imaging, NLP *= *natural language processing, OCAT *= *oculo-cognitive addition test, PET *= *positron emission tomography, PwPD *= *persons with Parkinson disease, QDA *= *quadratic discriminant analysis, RF *= *random forest, RS-fMRI *= *resting-state fMRI, sMRI *= *structural MRI, SVM *= *support vector machine, TMS *= *transcranial magnetic stimulation, VR *= *virtual reality, WMS *= *Wechsler Memory Scale. Reference numbers appear only in the study column.

Across the included literature, several broad methodological patterns emerged. Traditional machine-learning methods such as support vector machines (SVMs) and random forests^[5,9,14-43]^ were frequently applied in smaller or modality-specific datasets, whereas convolutional neural networks (CNNs)^[10,13,31,32,44-52]^ predominated in neuroimaging studies. More recent work increasingly incorporated transformer-based architectures and large language models,^[6,7,34,53,54]^ particularly in multimodal and speech-based applications.

The input data modalities were equally varied. Structural magnetic resonance imaging was the most common input, used in 34 studies.^[5,10,13,16,17,20,23,27,30,31,40,42,44,46,47,50-52,55-58]^ Functional neuroimaging – including resting-state fMRI, functional near-infrared spectroscopy (fNIRS), and PET – appeared in 13 studies.^[21,27,28,42,49,50,59]^ Neuropsychological evaluations and cognitive screening tests were used in 28 studies.^[4,8,11,14,15,19,29,35,40,41,43,45,51,56,60,61]^ Speech and language analysis was the primary input in 9 studies.^[6,18,26,53,54,62-64]^ Electronic health record (EHR) and clinical notes were used in 6 studies.^[7,24,31,38,41,65]^ Novel biomarkers and behavioral measures, including plasma proteomics,^[66]^ urine proteomics,^[30]^ electroencephalography (EEG)-derived connectivity,^[22,48,50]^ facial emotion analysis,^[67]^ eye movement features,^[68]^ TMS (transcranial magnetic stimulation),^[34]^ gait analysis,^[32]^ keystroke dynamics,^[69]^ cerebral blood flow (CBF),^[70]^ and digital cognitive tasks combined with wearable sensors^[25,71]^ – appeared in an emerging cluster of studies. Assessment of social cognition was also explored in a small feasibility study.^[12]^

The clinical settings varied widely. Speciality clinics and research centers accounted for the majority of the included studies (n = 38).^[12,16-18,20-23,26-30,34,37,39,40,42,44-58,60-63,69,70,72,73]^ Primary care or community screening settings were explicitly targeted in 22 studies.^[4,8,9,11,14,19,24,31-33,35,36,38,41,43,59,61,64,68,71]^ Several tools were designed for multiple settings or remote administration.^[7,13,25,65,74]^ Most studies focused on prodromal or early-stage MCI, several explicitly targeting preclinical stages.^[11,69]^ Overall, the literature suggests that modality choice, care setting, and study scale are major sources of heterogeneity and should be considered when interpreting reported diagnostic performance.

### 3.3. AI diagnostic performance for MCI

The overall diagnostic precision ranged from 62% to 100% in the included studies, reflecting substantial heterogeneity in AI methodology, input data modality, population characteristics, and validation approach. Table 2 summarizes the AI diagnostic performance by input data modality.

**Table 2:** Summary of AI diagnostic performance for MCI by input data modality.

Input modality	Representative AI methods	Accuracy range	Sensitivity range	AUC range	Clinical applicability
Structural MRI	SVM, CNN, DenseNet169, DAG 3D-CNN	85–99.7%	84–98.6%	0.92–1.00	Speciality clinic; requires imaging; high precision but resource intensive^[5,10,16,17,34,39-41,67]^
Functional neuroimaging (fMRI, fNIRS, PET)	LightGBM, CNN, SVM	82–96%	82–98%	0.88–0.99	Speciality/research; equipment-dependent^[21,27,28,36,37,56,68]^
Multimodal (imaging + cognitive + biomarker)	Transformer, ensemble, XGBoost	81–99.7%	90–100%	0.92–0.99	Speciality; highest overall precision^[7,33,34,37,45,50,51,53]^
Neuropsychological/ cognitive tests	ANN, RF, SVM, MLP	69–93.3%	74–100%	0.75–0.96	Primary care and speciality; accessible^[4,8,11,19,29,32,39,49,54,55,57-59,64]^
Speech & language	GPT-4, BERT, DNN, SVM	62–90%	62–90%	0.76–0.86	Primary/remote; minimal equipment^[6,18,26,44,47,48,60,69]^
EHR/clinical notes (NLP)	LASSO, hierarchical attention NN, gradient boosting	67–82%	2–80%	0.67–0.82	Primary care; uses existing data^[7,24,38,52,55,70]^
Plasma/urine biomarkers	Deep neural network, LASSO, SVM	95–99.5%	95–99%	0.97–1.00	Research/speciality; highest accuracy^[71,74]^
EEG-derived connectivity	Ensemble CNN, SVM	87–97.9%	87–98.4%	0.91–0.99	Speciality/research; requires a technician and equipment^[22,35,37]^
Behavioural sensors (gait, eye movement, VR)	SVM, KNN, LR, CNN	76–94.4%	76–100%	0.80–0.97	Primary/community; noninvasive; brief; scalable^[9,25,40,43,50,59,72,73]^

AI = artificial intelligence, AUC *= *area under the receiver operating characteristic curve, CNN *= *convolutional neural network, EHR *= *electronic health record, fMRI *= *functional magnetic resonance imaging, fNIRS *= *functional near-infrared spectroscopy, GPT *= *generative pretrained transformer, KNN *= *k-nearest neighbor, LASSO *= *least absolute shrinkage and selection operator, LR *= *logistic regression, MCI *= *mild cognitive impairment, MLP *= *multilayer perceptron, MRI *= *magnetic resonance imaging, NLP *= *natural language processing, PET *= *positron emission tomography, RF *= *random forest, SVM *= *support vector machine, VR *= *virtual reality.

### 3.4. Neuroimaging-based systems

Neuroimaging-based AI systems often reported the strongest performance estimates, particularly in structural MRI and multimodal imaging settings. A DAG 3D-CNN combined with SVM achieved 97.67% accuracy with 98.60% sensitivity from structural MRI.^[47]^ Transfer learning using DenseNet169 achieved 99.7% accuracy with AUCs ranging from 0.951 to 1.000.^[10]^ A voxel-based morphometry (VBM)-SVM approach achieved 90.5% accuracy in 1446 subjects.^[5]^ Additional studies demonstrated robust performance using 2D CNN,^[44]^ ensemble classifiers,^[16]^ SVM,^[17,20]^ multilayer perceptron,^[55]^ XGBoost,^[23,30,42,57]^ CNN with deep metric learning,^[51]^ and automated MRI-based classification.^[52,58]^ PET- and functional imaging-based methods also produced strong diagnostic performance in specialized settings.^[21,27,28,42,49,59]^ However, these high performance estimates were concentrated largely in internally validated studies and in research-enriched samples rather than routine clinical populations.

### 3.5. Multimodal approaches

Multimodal approaches integrating 2 or more data types consistently outperformed unimodal methods. Combining virtual reality-derived biomarkers with structural magnetic resonance imaging achieved 94.4% accuracy with 100% sensitivity,^[36]^ outperforming either modality alone. Integrating whole brain magnetic resonance radiomics, neuropsychological scores, and plasma biomarkers produced an accuracy of 81% and a macro-AUC of 0.92.^[37]^ A multimodal architecture integrating imaging and clinical data in 51,269 participants demonstrated robust performance on a scale.^[7]^ EEG connectivity analysis using ensemble CNNs achieved 97.90% precision with 98.40% sensitivity.^[48]^ Taken together, these studies suggest that multimodal integration may be one of the most promising directions for improving MCI diagnosis, although external validation remains limited.

### 3.6. Cognitive and behavioral assessment systems

AI systems applied to cognitive testing and behavioral assessment showed more moderate but potentially more deployable performance. A virtual kiosk test combining hand and eye movement analysis with SVM classification achieved 93.3% accuracy, 100% sensitivity, and 83.3% specificity.^[9]^ Other systems using questionnaires, computerized cognitive testing, and structured clinical data typically reported accuracy in the 70% to 85% range.^[11,14,19,24,35,41,43,61]^ Speech and language-based tools also demonstrated clinically interesting performance, especially for remote screening.^[6,18,26,53,54,62-64]^ These lower-burden approaches may offer greater practical accessibility than advanced imaging-based systems, despite somewhat lower peak performance in many studies.

### 3.7. Novel biomarker approaches

Several studies explored plasma proteomics,^[66]^ urine proteomics,^[73]^ EEG,^[48]^ gait analysis,^[32]^ facial emotion analysis,^[67]^ and eye movement-based tools.^[68]^ These approaches showed promising results but remain relatively early-stage and heterogeneous. At present, their main value lies in expanding the range of potential MCI indicators rather than providing a settled diagnostic standard.

### 3.8. Validation approaches and their impact on performance estimates

The validation methodology had a substantial and consistent effect on the reported performance. Studies employing only internal cross-validation reported higher accuracy than those employing external validation with independent datasets – illustrated by the 10% drop observed in 1 study.^[59]^ Small single-center studies – some with fewer than 30 participants^[11,12,20,58]^ – provide insufficient evidence for confidence in performance claims. Large-scale studies with diverse populations demonstrated more stable performance.^[7,13,15,61]^ Thus, reported diagnostic accuracy must be interpreted in the context of validation strategy and sample representativeness rather than as a modality-independent indicator of clinical readiness.

### 3.9. Direct comparison with physician diagnostic performance

Only 8 of the 71 included studies (11.3%) provided direct comparative data between AI and physician diagnostic performance for MCI. These studies offered consistent evidence that AI systems match or exceed the precision of physician diagnostics.^[4-7,26,60,72]^ Table 3 summarizes the key findings from all 8 comparative studies. These studies suggested that AI systems often performed comparably to or better than physicians in selected tasks, especially in sensitivity, but the studies differed substantially in physician comparator group, modality, patient population, and outcome definition.

**Table 3:** Summary of studies directly comparing AI and physician diagnostic performance for MCI.

Study	AI system	Physician type	AI performance	Physician performance	Key finding
[4]	Counterpropagation ANN	2 FPs, 1 neurologist, 1 geriatrician	Sensitivity 90.0%, Specificity 84.78%, AUC 95.2%	Collective sensitivity 46.66%, specificity 91.3%	AI demonstrated markedly superior sensitivity; Family physicians performed worst
[5]	SVM (BAAD/VSRAD)	2 neuroradiologists	Accuracy 90.5%	Accuracy 57.5% and 70.0%	AI outperformed both radiologists; kappa 0.35 0.56 with AI assistance but remained inferior
[6]	GPT-4	3 junior neurologists	Accuracy 81%, sensitivity 77%, specificity 83%	Accuracy 41–49%	GPT-4 significantly outperformed junior neurologists (*P* < .001)
[7]	Multimodal transformer	Neurologists (unassisted)	AI assistance: +26.25% AUROC	Unassisted neurologist AUROC	Significant AUROC improvement of 26.25% (*P* < .05); strongest evidence for collaborative model
[58]	PredictAD decision support	Clinicians (specialty care)	71% precision, 75% sensitivity, 68% specificity	Clinical criteria + biomarkers: significantly inferior (*P* ≤ .037)	AI-assisted diagnosis significantly outperformed biomarker-only and clinical criteria approaches
[42]	Ensemble SVM (memory clinic)	Clinicians (MCI staging)	PPV 91%, Cohen κ = 0.81	Clinician staging (reference)	High AI–clinician agreement (κ = 0.81); 91% PPV for causal hypotheses
[26]	SVM, Extra Trees, RF (speech AI)	Standardised neurocognitive assessment	AUC 0.76–0.86	Standardised neuropsych scores (reference)	Speech AI demonstrated moderate-to-good discrimination; supports remote screening feasibility

AI = artificial intelligence, ANN *= *artificial neural network, AUROC *= *area under receiver operating characteristic curve, FP *= *family physician, LR *= *logistic regression, MCI *= *mild cognitive impairment, PPV *= *positive predictive value, RF *= *random forest, SVM *= *support vector machine, VBM *= *voxel-based morphometry.

The most detailed comparison was conducted by Araujo et al in a primary care population of 128 MCI cases and 203 controls.^[4]^ A counterpropagation ANN achieved 90.0% sensitivity and 84.78% specificity (AUC 95.2%), compared with the collective 46.66% sensitivity and 91.3% specificity of the physician panel. Syaifullah et al compared a VBM-based SVM system with 2 neuroradiologists reviewing the same structural MRI scans in 1446 subjects.^[5]^ The AI system achieved 90.5% accuracy, whereas the neuroradiologists achieved 57.5% and 70.0%, respectively. Yang et al compared GPT-4 with 3 junior neurologists using language samples from 174 participants,^[6]^ and GPT-4 achieved 81% accuracy compared with 41 to 49% for the neurologists. Xue et al reported that AI assistance improved neurologist evaluation by approximately 26.25% in AUROC in a large multimodal dataset.^[7]^ Other studies similarly suggested favorable AI or AI-assisted performance in selected contexts.^[26,60,72]^

Overall, the comparative evidence is promising but limited. Because only a small number of direct comparison studies are available and these studies are methodologically heterogeneous, the current literature supports cautious interpretation rather than a definitive claim that AI is uniformly superior to physicians in diagnosing MCI.

### 3.10. Clinical implementation considerations

Test duration was a major differentiating factor between AI systems. Computerized cognitive tests required approximately 5 minutes of administration,^[19,41]^ substantially shorter than traditional neuropsychological batteries that may require 40 minutes or more.^[9]^ The eye movement-based assessment required less than 1 minute.^[68]^ Speech recording and automated analysis required only brief samples.^[6,26,54,64]^

Cost-effectiveness has been explicitly addressed in few studies. One study estimated that AI-assisted decision support could save $1500 to $2200 per patient annually.^[72]^ Studies using EHR data or structured cognitive tools emphasized relatively low marginal implementation costs.^[7,24,38,41,65]^ By contrast, approaches requiring advanced neuroimaging,^[10,47]^ specialized EEG equipment,^[48,50]^ or virtual reality systems^[36]^ may present practical resource barriers.

A recurring theme was the trade-off between performance and accessibility. The highest reported accuracy estimates were often associated with specialized imaging or biomarker platforms, whereas systems using cognitive assessments, speech samples, or routine clinical data were more feasible for broad screening or primary care implementation, albeit typically with lower reported performance.

## 4. Discussion

This scoping review synthesized evidence from 71 primary research studies on AI-based diagnosis of MCI. Several clinically relevant themes emerged.

First, many AI systems reported high diagnostic accuracy for MCI across diverse methodologies and data modalities, but performance varied considerably by input modality, validation strategy, and study context. Multimodal approaches that combined neuroimaging, cognitive assessments, and biomarkers often reported the strongest results.^[7,13,36,37]^ However, these findings should be interpreted cautiously because some of the most striking estimates came from internally validated, single-center, or highly selected samples.

Second, direct comparisons between AI and physicians remain remarkably scarce. Only 8 of the 71 included studies (11.3%) provided head-to-head data. In these limited and heterogeneous studies, AI systems generally matched or exceeded physician performance, especially in sensitivity, but the current evidence base is not large enough to support broad generalizations across clinical settings, physician groups, or diagnostic modalities.^[4-7,26,60,72]^

Third, the most clinically compelling model supported by the current evidence is AI as a decision-support tool that complements rather than replaces clinician judgment. One large study reported an approximately 26% improvement in AUROC when neurologists used AI assistance,^[7]^ suggesting that AI-supported assessment may improve diagnostic performance in some contexts. However, this finding should be interpreted as an important example rather than a universally established clinical effect.

**Figure 2.**
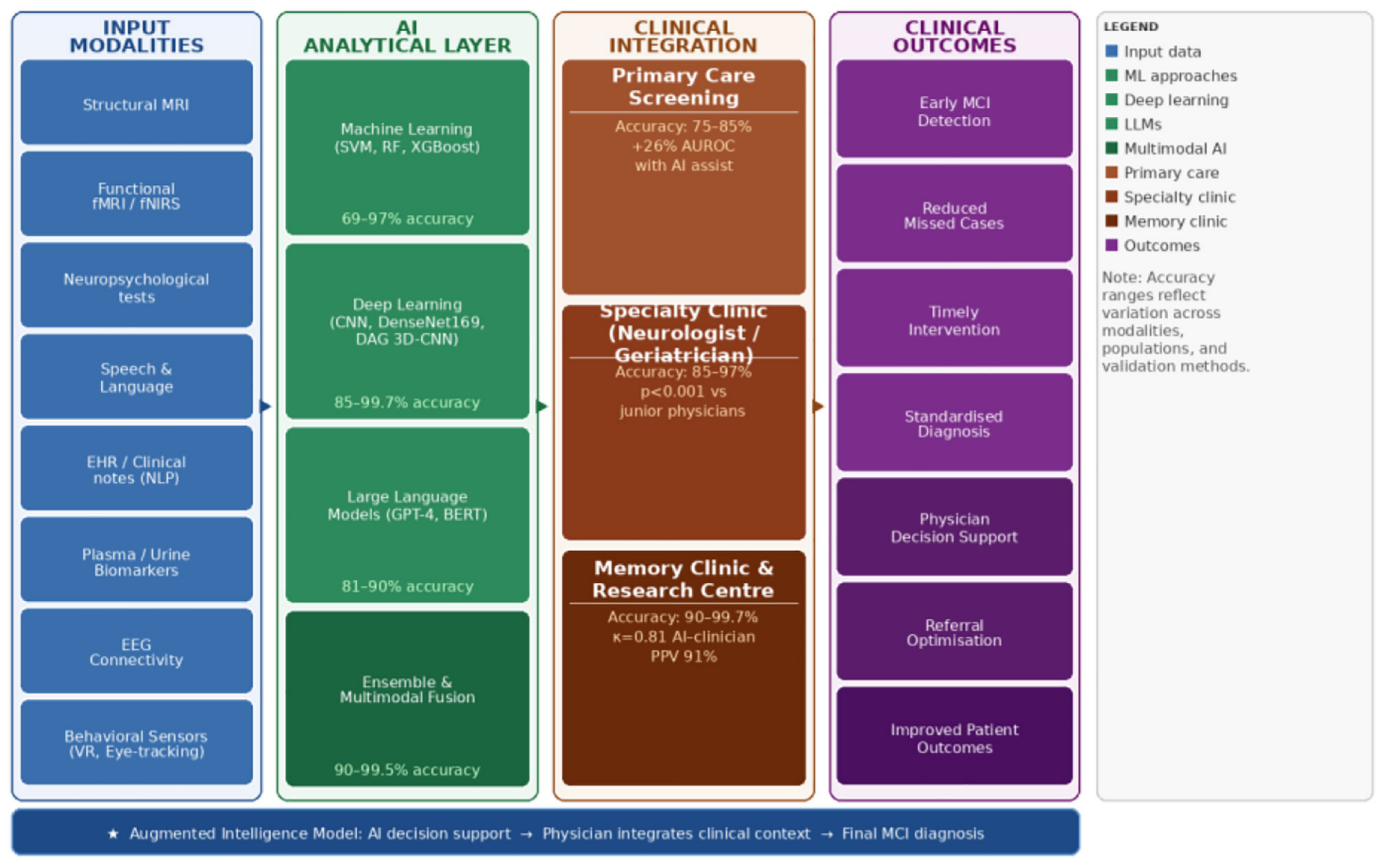
Conceptual framework for AI-augmented physician diagnosis in clinical settings study. AI = artificial intelligence, CNN = convolutional neural network, EEG = electroencephalography, EHR = electronic health record, fMRI = functional magnetic resonance imaging, fNIRS = functional near-infrared spectroscopy, MCI = mild cognitive impairment, NLP = natural language processing, RF = random forest, SVM = support vector machine, VR = virtual reality.

The heterogeneity of reported performance reflects real differences in clinical context, patient population, input modality, and validation design. A modality-context framework may therefore be more useful than a simple ranking of algorithms. Neuroimaging-based AI, often reporting accuracy in the 85% to 99% range, appears best suited to specialty or research settings.^[5,10,16,17,44,47,55]^ Cognitive and behavioral AI systems, often reporting more moderate accuracy, may offer more scalable deployment in primary care or community screening.^[8,11,14,19,24,35,41,43,61]^ Speech- and EHR-based systems occupy an intermediate space, with lower barriers to implementation but variable performance.^[26,38,64,65]^ Figure 2 illustrates the conceptual framework for AI-augmented physician diagnosis in clinical settings. These patterns are broadly consistent with recent scoping and systematic reviews of AI for MCI detection and AI-versus-clinician diagnosis.^[75-77]^

### 4.1. Methodological considerations

The validation approach had a substantial and consistent effect on the reported performance. External validation generally produced lower accuracy than internal cross-validation, as illustrated by the 10-percentage-point drop observed in 1 study.^[59]^ Systems reporting near-perfect performance^[10,47,48,66]^ therefore require especially cautious interpretation. Sample size and representativeness also influenced the stability of findings. Large, multisite studies demonstrated more stable performance,^[7,13,15,61]^ whereas very small single-center studies provide limited confidence in generalizability.^[11,12,20]^

### 4.2. Clinical implications

For practising clinicians, AI decision-support tools may offer the greatest value when integrated into existing diagnostic workflows rather than used as autonomous systems. Primary care may benefit most from brief, accessible screening tools,^[4,8,9,11,14,19,24,35,41,43,61,68,71]^ while neurologists and geriatricians may benefit more from multimodal decision-support platforms.^[7,13,29,32,36,37,39,46,56,72,74]^

### 4.3. Limitations

Although the search was conducted across 6 major bibliographic databases with a comprehensive Boolean strategy, gray literature and non-English publications were not systematically searched, which may have introduced publication and language bias. No formal risk-of-bias assessment was conducted for individual studies. The absence of meta-analytic synthesis limits the precision of pooled performance interpretation. The scarcity of direct physician-comparison studies is itself a major limitation of the evidence base. Eight studies are insufficient to draw robust conclusions about the relative performance of AI versus physicians across diverse settings. The predominance of retrospective designs, internal-only validation, short follow-up periods, and single-center recruitment further constrains generalizability.

### 4.4. Future directions

The evidence base supports several priorities for future research. First, prospective studies that directly compare AI diagnostic performance with that of multiple physician groups are urgently needed. Second, external validation of high-performance AI systems^[10,47,48,66]^ in independent and demographically representative populations is a prerequisite for wider clinical deployment. Third, health economic analyses are needed to compare AI-augmented and unaugmented diagnostic pathways. Fourth, implementation research should examine clinician acceptance, workflow integration, and patient-centered outcomes. Finally, standardized reporting guidelines for AI diagnostic accuracy studies would improve interpretability and clinical utility.

## 5. Conclusion

This scoping review indicates that many AI systems have reported promising diagnostic performance for MCI across diverse methodologies and data modalities. In the limited available direct comparisons, AI systems generally matched or exceeded physician performance, although the evidence base remains small and methodologically heterogeneous. Multimodal approaches combining neuroimaging, cognitive assessment, and biomarker data appear especially promising,^[7,36,37,46]^ while brief cognitive-task and speech-based approaches may offer more accessible options for primary care deployment.^[9,18,19,26,62,64]^ The paucity of direct comparative studies, the predominance of internally validated retrospective designs, and limited demographic diversity constrain confident conclusions about real-world clinical utility. Prospective validation studies with standardized physician benchmarking, external validation, and patient-centered outcome assessment are needed to establish the clinical utility and generalizability of AI-based MCI diagnosis.

## Author contributions

**Conceptualization:** Haewon Byeon.

**Data curation:** Haewon Byeon.

**Formal analysis:** Haewon Byeon.

**Funding acquisition:** Haewon Byeon.

**Investigation:** Haewon Byeon.

**Methodology:** Haewon Byeon.

**Project administration:** Haewon Byeon.

**Resources:** Haewon Byeon.

**Software:** Haewon Byeon.

**Supervision:** Haewon Byeon.

**Validation:** Haewon Byeon.

**Visualization:** Haewon Byeon.

**Writing – original draft:** Haewon Byeon.

**Writing – review & editing:** Haewon Byeon.


