# Supplementary material for: Artificial intelligence for early diagnosis of mild cognitive impairment: A scoping review comparing diagnostic accuracy with physician performance
Source: Medicine (Baltimore). 2026 Jul 31;105(31):e50006. doi: 10.1097/MD.0000000000050006 (PMC13433120; doi:10.1097/MD.0000000000050006)
Supplement: Supplementary file 1 [file medi-105-e50006-s001.docx]

Supplementary Appendix 1. Database-specific search strategy

Review title:

Artificial Intelligence for Early Diagnosis of Mild Cognitive Impairment: A Scoping Review Comparing Diagnostic Accuracy with Physician Performance

Search period:

January 1, 2000 to March 31, 2026

Language restriction:

English

Core concepts:

1) Mild cognitive impairment

2) Artificial intelligence / machine learning

3) Diagnosis / classification / screening / predictive performance

PubMed/MEDLINE

(("Mild Cognitive Impairment"[Mesh] OR "mild cognitive impairment"[tiab] OR MCI[tiab] OR "cognitive decline"[tiab] OR "prodromal Alzheimer*"[tiab])

AND

("Artificial Intelligence"[Mesh] OR "Machine Learning"[Mesh] OR "artificial intelligence"[tiab] OR "machine learning"[tiab] OR "deep learning"[tiab] OR "neural network*"[tiab] OR "artificial neural network*"[tiab] OR "convolutional neural network*"[tiab] OR CNN[tiab] OR "support vector machine*"[tiab] OR SVM[tiab] OR "random forest"[tiab] OR XGBoost[tiab] OR transformer*[tiab] OR "large language model*"[tiab] OR GPT-4[tiab] OR "natural language processing"[tiab] OR NLP[tiab])

AND

(diagnos*[tiab] OR detect*[tiab] OR screen*[tiab] OR classif*[tiab] OR predict*[tiab] OR accurac*[tiab] OR sensitiv*[tiab] OR specificit*[tiab] OR AUROC[tiab] OR AUC[tiab]))

Filters: English; 2000/01/01 to 2026/03/31.

EMBASE

('mild cognitive impairment'/exp OR 'mild cognitive impairment':ti,ab OR mci:ti,ab OR 'cognitive decline':ti,ab OR 'prodromal alzheimer*':ti,ab)

AND

('artificial intelligence'/exp OR 'machine learning'/exp OR 'artificial intelligence':ti,ab OR 'machine learning':ti,ab OR 'deep learning':ti,ab OR 'neural network*':ti,ab OR 'artificial neural network*':ti,ab OR 'convolutional neural network*':ti,ab OR cnn:ti,ab OR 'support vector machine*':ti,ab OR svm:ti,ab OR 'random forest':ti,ab OR xgboost:ti,ab OR transformer*:ti,ab OR 'large language model*':ti,ab OR 'natural language processing':ti,ab OR nlp:ti,ab)

AND

(diagnos*:ti,ab OR detect*:ti,ab OR screen*:ti,ab OR classif*:ti,ab OR predict*:ti,ab OR accurac*:ti,ab OR sensitiv*:ti,ab OR specificit*:ti,ab OR auroc:ti,ab OR auc:ti,ab)

AND [english]/lim AND [2000-2026]/py

Web of Science Core Collection

TS=(("mild cognitive impairment" OR MCI OR "cognitive decline" OR "prodromal Alzheimer*")

AND

("artificial intelligence" OR "machine learning" OR "deep learning" OR "neural network*" OR "artificial neural network*" OR "convolutional neural network*" OR CNN OR "support vector machine*" OR SVM OR "random forest" OR XGBoost OR transformer* OR "large language model*" OR "natural language processing" OR NLP)

AND

(diagnos* OR detect* OR screen* OR classif* OR predict* OR accurac* OR sensitiv* OR specificit* OR AUROC OR AUC))

Refined by: Document type = Article; Language = English; Timespan = 2000-2026.

Scopus

TITLE-ABS-KEY(("mild cognitive impairment" OR MCI OR "cognitive decline" OR "prodromal Alzheimer*")

AND

("artificial intelligence" OR "machine learning" OR "deep learning" OR "neural network*" OR "artificial neural network*" OR "convolutional neural network*" OR CNN OR "support vector machine*" OR SVM OR "random forest" OR XGBoost OR transformer* OR "large language model*" OR "natural language processing" OR NLP)

AND

(diagnos* OR detect* OR screen* OR classif* OR predict* OR accurac* OR sensitiv* OR specificit* OR AUROC OR AUC))

AND PUBYEAR > 1999 AND PUBYEAR < 2027 AND (LIMIT-TO(LANGUAGE, "English")) AND (LIMIT-TO(DOCTYPE, "ar"))

PsycINFO

((DE "Mild Cognitive Impairment" OR TI("mild cognitive impairment" OR MCI OR "cognitive decline" OR "prodromal Alzheimer*") OR AB("mild cognitive impairment" OR MCI OR "cognitive decline" OR "prodromal Alzheimer*"))

AND

(DE "Artificial Intelligence" OR DE "Machine Learning" OR TI("artificial intelligence" OR "machine learning" OR "deep learning" OR "neural network*" OR "artificial neural network*" OR "convolutional neural network*" OR CNN OR "support vector machine*" OR SVM OR "random forest" OR XGBoost OR transformer* OR "large language model*" OR "natural language processing" OR NLP) OR AB("artificial intelligence" OR "machine learning" OR "deep learning" OR "neural network*" OR "artificial neural network*" OR "convolutional neural network*" OR CNN OR "support vector machine*" OR SVM OR "random forest" OR XGBoost OR transformer* OR "large language model*" OR "natural language processing" OR NLP))

AND

(TI(diagnos* OR detect* OR screen* OR classif* OR predict* OR accurac* OR sensitiv* OR specificit* OR AUROC OR AUC) OR AB(diagnos* OR detect* OR screen* OR classif* OR predict* OR accurac* OR sensitiv* OR specificit* OR AUROC OR AUC)))

Limiters: Peer-reviewed journal article; English; 2000-2026.

CINAHL

((MH "Mild Cognitive Impairment" OR TI "mild cognitive impairment" OR AB "mild cognitive impairment" OR TI MCI OR AB MCI OR TI "cognitive decline" OR AB "cognitive decline")

AND

(MH "Artificial Intelligence" OR MH "Machine Learning" OR TI "artificial intelligence" OR AB "artificial intelligence" OR TI "machine learning" OR AB "machine learning" OR TI "deep learning" OR AB "deep learning" OR TI "neural network*" OR AB "neural network*" OR TI "support vector machine*" OR AB "support vector machine*" OR TI "random forest" OR AB "random forest" OR TI XGBoost OR AB XGBoost OR TI transformer* OR AB transformer* OR TI "natural language processing" OR AB "natural language processing")

AND

(TI diagnos* OR AB diagnos* OR TI detect* OR AB detect* OR TI screen* OR AB screen* OR TI classif* OR AB classif* OR TI predict* OR AB predict* OR TI accurac* OR AB accurac* OR TI sensitiv* OR AB sensitiv* OR TI specificit* OR AB specificit* OR TI AUROC OR AB AUROC OR TI AUC OR AB AUC))

Limiters: English Language; Published Date 20000101-20260331; Peer-reviewed.

Supplementary search procedures

- Reference lists of all included studies and relevant reviews were hand-searched.

- Duplicates were removed before title/abstract screening.

- Conference abstracts without sufficient methodological detail, editorials, case reports, systematic reviews, and meta-analyses were excluded.
